# Governing the resilience of neoliberalism through biopolitics

**DOI:** 10.1177/1354066116676321

**Published:** 2016-11-21

**Authors:** Luca Mavelli

**Affiliations:** University of Kent, UK

**Keywords:** Biopolitics, Foucault, governmentality, Greek sovereign debt crisis, neoliberalism, resilience

## Abstract

Neoliberalism is widely regarded as the main culprit for the 2007/2008 global financial crisis. However, despite this abysmal failure, neoliberalism has not merely survived the crisis, but actually ‘thrived’. How is it possible to account for the resilience of neoliberalism? Existing scholarship has answered this question either by focusing on the distinctive qualities of neoliberalism (such as adaptability, internal coherence and capacity to incorporate dissent) or on the biopolitical capacity of neoliberalism to produce resilient subjects. This article adopts a different perspective. Drawing on and partially challenging the perspective of Michel Foucault, I argue that neoliberalism and biopolitics should be considered two complementary governmental rationalities, and that biopolitical rationalities contribute to governing the uncertainties and risks stemming from the neoliberalization of life. Biopolitics, in other words, plays a key role in governing the resilience of neoliberalism. Through this conceptual lens, the article explores how biopolitical rationalities of care have been deployed to govern the neoliberal crisis of the Greek sovereign debt, which threatened the stability of the European banking system and, I shall argue, the neoliberal life, wealth and well-being of the European population. The article discusses how biopolitical racism is an essential component of the biopolitical governance of neoliberalism. Biopolitical racism displaces the sources of risk, dispossession and inequality from the neoliberal regime to ‘inferior’ populations, whose lack of compliance with neoliberal dictates is converted into a threat to our neoliberal survival. This threat deserves punishment and authorizes further dynamics of neoliberal dispossession.

## Introduction

It has been widely observed that the 2007/2008 global financial crisis engendered the expectation of a shift towards a ‘post-neoliberal regime’ that ultimately failed to materialize (Konings, 2016: 268; see also Crouch, 2011; Dean, 2014; Harvey, 2011; Peck, 2010; Peck et al., 2010; Schmidt and Thatcher, 2013). The main dimensions of neoliberalism — competition, privatization, deregulation, individual responsibility, privatization of profits and socialization of losses, the centrality of the market mechanism for the allocation of essential goods and services, and (specifically in the case of Europe) austerity — have retained, if not augmented, their ideological prominence and practical relevance. Irrespective of how neoliberalism is understood — as a political philosophy, governmental rationality, economic theory or regime of subjectification (Dean, 2014: 151) — its hegemony seems unchallenged. Scholars have questioned this apparent anomaly; after all, all major financial crises, such as the 1930s’ Great Depression and the 1970s’ crisis of Keynesianism, have been ‘turning points for economic shifts and public policies’ (Bohle and Greskovits, 2015: 1). Others, however, have highlighted the intimate connection between neoliberalism and crisis (Harvey, 2011; Mirowski, 2013; Peck et al., 2010; Roitman, 2013), namely, how neoliberalism as a response to the 1970s’ crisis of Keynesianism has resulted in a socio-political and economic paradigm routinely punctuated by crises. Yet, even the proponents of the idea of neoliberalism as a crisis-driven mode of governance consider that the magnitude and scale of the 2007/2008 global financial crisis, which has triggered the worst recession since the Great Depression, has been such that it would not have been unreasonable to expect a more substantial challenge to the neoliberal paradigm. Why has this not happened? How is it possible to account for the resilience of neoliberalism? This is the main question that this article will strive to address.

Two types of scholarship have directly or indirectly tackled this question. A first group of scholars has focused on the distinctive qualities of neoliberalism. In particular, they have emphasized: the amorphous and adaptable nature of neoliberalism (Schmidt and Thatcher, 2013), which often results in tensions between neoliberal theory and practice (Harvey, 2005, 2011); neoliberalism’s parasitical relationship with existing social formations (Peck et al., 2010); its being a form of ‘thought collective’, namely, an intellectual framework capable of absorbing dissent and crystallizing consensus (Dean, 2014) by offering simple solutions to complex problems (Schmidt and Thatcher, 2013); its being the carrier of the interests of large corporations, which have come to dominate public life (Crouch, 2011); and its internal coherence and capacity to reorganize through the incorporation of uncertainty and risk (Konings, 2016). All these factors conjure up the absence of a strong ideological opponent, which leaves neoliberalism virtually unchallenged (Peck et al., 2010; Schmidt and Thatcher, 2013).

A second group of scholars has explained the resilience of neoliberalism by focusing on its biopolitical dimension. Their argument is that neoliberalism should be primarily understood as a regime of subjectification geared towards the production of resilient subjects capable of adapting to the neoliberal mechanisms of production, exploitation, accumulation and dispossession (Chandler and Reid, 2016; Dean, 2014; Joseph, 2013; Reid, 2012; Schmidt, 2015). The resilience of neoliberalism would thus rest on the resilience of its subjects and their capacity to withstand the shocks of a socio-economic order naturally attuned to produce crises. This perspective generally draws on Michel Foucault’s (2010) understanding of neoliberalism as a regime of subjectification revolving around competition. It considers biopolitics, the politics of ‘making live and letting die’ (Foucault, 1978, 2003), as a function of the broader neoliberal order, in line with Foucault’s argument that neoliberalism should be understood as ‘the general framework of biopolitics’ (Foucault, 2010: 22n; see also Vatter, 2014: 163).

My goal in this article is to provide an alternative account of the resilience of neoliberalism. Those perspectives that focus on its distinctive qualities (adaptability, capacity to incorporate dissent, internal coherence, etc.) implicitly assume that neoliberalism is the dominant form of modern governmental rationality. On the other hand, those perspectives that focus on the capacity of neoliberalism to produce resilient subjects reduce biopolitics to a dimension of neoliberalism. My contention is that modern governmentality — the ensemble of meanings, significations and practices actively deployed to govern populations — encompasses two complementary and mutually reinforcing forms of rationality: neoliberalism and biopolitics. Whereas neoliberalism rests on the principles of competition, inequality and ‘governing for the market’ (Foucault, 2010), biopolitics relies on logics of care of the population and racism towards those who threaten its survival and well-being (Foucault, 2003). Moving from this perspective, I argue that the resilience of neoliberalism should be accounted for as a product not just of the distinctive qualities of neoliberalism, including its capacity to produce resilient subjects, but also of biopolitical rationalities that contribute to regulate, modulate and govern the uncertainties and risks stemming from the neoliberalization of life, and, in particular, neoliberalism’s inclination to produce crises. I will thus argue that biopolitics cannot be reduced to a dimension of neoliberalism. Biopolitical governmental rationalities govern crises and ensure the resilience of the neoliberal project through technologies of care of the population and biopolitical racism that partially offset neoliberal dynamics by projecting their cost onto ‘inferior’ populations. An exclusive focus on neoliberal rationalities overlooks how governing the resilience of neoliberalism entails intervening on the biological continuum of life by dividing human beings into competing species/races and normalizing the view that the death of ‘the inferior race … will make life in general healthier’ (Foucault, 2003: 255).

In order to advance these arguments, the discussion proceeds in three main steps. First, I provide a brief overview of existing accounts of the resilience of neoliberalism and emphasize how they either neglect biopolitics or confine it to a dimension of the neoliberal order. Second, drawing on and partially challenging Michel Foucault, I discuss how neoliberalism and biopolitics may be considered two complementary governmental rationalities and how biopolitical rationalities of care and racism contribute to governing the growing risk and uncertainty stemming from the neoliberalization of life. Third, I explore how biopolitical rationalities of care and racism have been deployed to govern the neoliberal crisis of the Greek sovereign debt and further promote the neoliberal financialized life of the European population.

This analysis aims to contribute to three interrelated growing fields of research, namely, cultural and critical international political economy, the resilience of neoliberalism, and the study of neoliberalism and biopolitics. To this end, it strives to articulate a novel perspective to analyse the relationship between neoliberalism and biopolitics and develop an alternative interpretation of the governmentality of the Greek debt crisis. Indirectly, this article can also be read as a contribution to governmentality studies. This heterogeneous body of scholarship has either understood biopolitics as a dimension of neoliberalism and neglected the importance of biopolitical racism as an autonomous source of governmental rationality (see, for instance, Burchell et al., 1991; Miller and Rose, 2008; Bröckling et al., 2010), or acknowledged that biopolitics cannot ‘be reduced to the logics of a particular liberal governmentality’ (Nadesan, 2008: 4; see also Dean, 2010: 120), but nonetheless paid very limited attention to biopolitical racism. Although I do not explicitly engage with this literature — which has not specifically tackled the question of the resilience of neoliberalism — I will emphasize the importance of national boundaries and biopolitical racism in governing ‘the economic fates of citizens within a national territory’ (Miller and Rose, 2008: 89). In particular, I will argue that governing ‘the lives of individuals in liberal societies’ entails not just nurturing neoliberal mechanisms of competition as frameworks of self-government in a new economic landscape in which the economy is no longer ‘co-extensive’ with the boundaries of a nation-state (Miller and Rose, 2008: 89, 95), but also caring for the population and drawing the line between who must live and who must die according to logics of biopolitical racism within, but possibly more importantly *across*, national boundaries.

## The resilience of neoliberalism: Existing explanations

Neoliberalism has been variously defined as an economic theory, a political philosophy, an ideology, a socio-political project and a governmental rationality characterized by a heterogeneous set of ideas, practices and regimes of power. Neoliberal ideas include: a belief in the superior capacity of markets over states to create and allocate wealth; the view that states should be scaled back in order to free up markets; the notion that growth can be promoted through competition, privatization, deregulation and financialization; a belief in the individual as a source of human capital, and in inequality as a reflection of one’s endowment of human capital and as a force which can promote entrepreneurship; and the view that all relations can be analysed and measured in economic cost/benefit terms. These ideas have translated into a varied set of practices, such as fiscal discipline, austerity, debt conditionality, structural adjustments, the removal of trade barriers, the commodification of goods and services, the privatization of profits and socialization of losses, the retrenchment of the welfare state and privatization of welfare provisions, the financialization of the economy and everyday life, labour flexibility, and the race to the bottom in labour standards. These practices have resulted in multiple regimes of power geared towards the restoration of class power through the progressive dismantlement of social provisions, mechanisms of upward redistribution, dynamics of accumulation and dispossession, the exploitation of labour, the extraction of surplus from life, and the transfer of wealth from the periphery to the centre, and from the ‘South’ to the ‘North’.

Irrespective of how neoliberalism is understood, its ideas and practices — particularly financialization and deregulation — have been widely regarded as the main culprits of the 2007/2008 global financial crisis. However, despite this abysmal failure, neoliberalism has not ‘merely survived’ the crisis, but actually ‘thrived’ (Schmidt and Thatcher, 2013: xvi). In order to account for this puzzling resilience, existing scholarship has pursued two main routes of inquiry. A first group of scholars has focused on the distinctive qualities of neoliberalism, and, in particular, on ‘the generality, diversity, and mutability of neo-liberal ideas’, which have often resulted in gaps between neoliberal rhetoric and practice (Schmidt and Thatcher, 2013: xix). A second group of scholars has focused on the biopolitical capacity of neoliberalism to produce resilient subjects. In the remainder of this section, I provide a brief overview of these two perspectives and introduce the key argument of this article, namely, that these approaches, although extremely insightful, either fail to problematize the role of biopolitics or fail to conceptualize it as a complementary governmental rationality that plays a key role in governing the resilience of neoliberalism.

For Vivien Schmidt and Mark Thatcher (2013: 27), the amorphous, protean and adaptable nature of neoliberalism has been essential for its resilience. Neoliberalism has unashamedly progressed ‘from hostility against the state to the desire for a strong state’, from deregulation to re-regulation, ‘from passive reduction of social spending and job protections to active use of welfare to promote market efficiency’. Similarly, Jamie Peck (2010: 106) highlights how neoliberalism has shifted ‘from structural adjustment to good governance, from budget cuts to regulation-by-audit … from privatization to public–private partnership, from greed-is-good to markets-with-morals’. These multiple and contradictory manifestations have led many scholars to question the very notion of neoliberalism and the extent to which it may have become a buzzword that, in the attempt to capture under a single conceptual lens many disparate phenomena, has been ‘stretched too far to be productive as a critical analytical tool’ (Clarke, 2008: 135). However, as Peck, Theodore and Brenner (2010) suggest, the strength of neoliberalism lies precisely in its capacity to adapt and colonize different social environments. Accordingly, they argue that neoliberalism should be understood as an incomplete socio-political project that ‘exists in an essentially parasitical relationship with those extant social formations with which it has an antagonistic relationship, such as state socialism, social democracy, or neoconservative authoritarianism’ (Peck et al., 2010: 104). The result is that there is no clear separation between neoliberalism and its opponents. From this perspective, the resilience of neoliberalism reflects the latter’s capacity to change, adapt and co-opt different and often contending ideas and social formations, thus turning itself into an elusive target for opposing forces.

Several scholars have pointed out how the variable and adaptable nature of neoliberalism often translates into a fundamental incoherence between its theoretical assumptions and practical implementation, which conceals neoliberalism’s oppressive and exploitative nature. Marxist scholar David Harvey (2010: 10) has discussed how ‘[o]ne of the basic pragmatic principles that emerged in the 1980s’, namely, the idea that ‘state power should protect financial institutions at all costs’, blatantly contradicts the notions of non-interference and self-regulating markets prescribed by orthodox neoliberalism. Similarly, Colin Crouch (2011: viii) has stressed the tension between the neoliberal advocacy of the competitive market as the most efficient system for the production and allocation of wealth, and the neoliberal implementation of a regime of governance that has favoured the monopolistic ‘dominance of public life by the giant corporation’. Philip Mirowski (2013) has advanced a complementary critique. Although neoliberal advocates have regularly targeted the state as a bureaucratic burden that compresses individual freedoms and distorts the efficient functioning of the market, their ‘primary ambition … is to redefine the shape and functions of the state, not to destroy it’ (Mirowski, 2013: 48). A strong state is essential to uphold, defend and guarantee the existence and functioning of the market.

What Harvey’s, Crouch’s and Mirowski’s critiques share is the proposition that the neoliberal gap between theory and practice has enabled neoliberal ideas and their carriers, that is, economic and financial elites, to ‘capture’ part of the media, intellectuals, political parties and, ultimately, state power. This perspective is central to post-Keynesian and Marxist approaches to the ‘governance of the global financial crisis’ (Langley, 2015: 19) and the resilience of neoliberalism. It is well summarized by Harvey’s (2010: 115) argument that neoliberal ‘capitalism has to create external powers in order to save itself from its own internal contradictions’, namely, its innate tendency to over-accumulation, speculation and crisis. The state has therefore been ‘captured’, that is, turned into the tool of the ruling financial elites in order to ensure the resilience of neoliberalism.

Martijn Konings (2016: 269) has questioned this ‘capture model’ and argued that the fact that the 2008 crisis has resulted in ‘relatively few restrictive regulations on financial institutions’ is a product of neoliberalism’s capacity to frame political action as inherently speculative. Konings’s view deserves particular attention because, unlike the aforementioned perspectives, it employs a Foucauldian approach. As I shall discuss in greater detail in the next section, according to Foucault, and unlike the scholars so far discussed, neoliberalism entails not the domination of the market over politics, but the economization of the state, that is, the idea that the state should be ruled, modelled and governed as a large corporation. For Konings, the behaviour of the neoliberal state should be analysed through the lenses of Friedrich Hayek’s neoliberal theory. Hayek considered that the state could not engineer social order since it could not ‘place itself outside the logic of risk and speculation’ (Konings, 2016: 278); therefore, order could only emerge as the spontaneous result of self-regulating market forces. Konings (2016: 277) discusses how the USA’s attempt to regulate ‘banks’ money-creating abilities’ resulted in new forms of ‘shadow banking’ that circumvented the regulatory framework. This suggests that governments cannot prevent the occasional failures of the market, but can manage market crises by converting ‘uncertainty into unambiguous authority’ (Konings, 2016: 273).

Accordingly, the governments’ bailout of financial institutions hit by the crisis and deemed ‘too big to fail’ should be understood not as inconsistent with neoliberal assumptions and as the product of ‘captured’ states, but as a result of the subjectification of states into ‘highly speculative’ actors. Governmental institutions, Konings (2016: 274) maintains, have not been colonized and hetero-directed by financial elites to purchase their toxic assets ‘whose value was fundamentally in doubt’, but have embraced the ‘only possible course of action’: protecting ‘the nodal points of the financial system’, that is, the banks, in order to safeguard their existence, that is, the existence of the market, which is the primary and fundamental task of states in a neoliberal order. This argument highlights an important difference between Harvey’s Marxist approach and Konings’s Foucauldian approach. Whereas Harvey considers neoliberalism as the ‘retreat’ of politics and ‘domination of the market’ (see Lemke, 2003: 177) and explains the resilience of neoliberalism as the result of ‘captured states’ saving the market, Konings deems neoliberalism as a process of economization of state and society and accounts for the resilience of neoliberalism as the product of ‘economized states’ acting in a context of uncertainty that turns states into speculative agents and encourages them to adopt a decisionist — ‘Schmittian’ — understanding of sovereign power.

Konings’s perspective has the merit of overcoming the traditional Marxist divide between states and markets. Nonetheless, his approach shares with Harvey and the other scholars so far discussed an underlying neglect of the role played by biopolitical rationalities in the resilience of neoliberalism. This neglect is particularly evident in Konings’s almost ‘technical’ account. Working within the framework of the economization of state and society (hence beyond the idea of the market oppressing the political and social life of the individual), but without considering the biopolitical implications of this economization, leads Konings to reduce the question of the resilience of neoliberalism to a problem of ‘governing the system’ in a complex framework of uncertainty. This raises questions about how the resilience of neoliberalism may impact on the life/biopolitical life of the population and, more broadly, about the role that biopolitics may play in the resilience of neoliberalism.

A growing group of scholars has tackled these questions by analysing neoliberalism as a biopolitical regime of subjectification mired in the production of resilient subjects. The notion of resilience has its origin in the 1970s’ ecology literature and accounts for the capacity of complex ecosystems to withstand and adapt to threats, shocks and crises that may undermine their existence (Evans and Reid, 2013; Walker and Cooper, 2011). Over the last few years, this concept has received increasing attention in the International Relations literature on crisis management. In particular, numerous scholars have explored how resilience is becoming a defining feature of a neoliberal governmental regime that is progressively shifting from equilibrium to adaptation. According to Mitchell Dean (2014), this shift is a product of a qualitative transformation of neoliberalism. Marked by a relentless pattern of crises, poor economic growth and growing inequality, neoliberalism is casting off ‘its supposition of economic equilibrium and its triumphalist narratives of the welfare-generating properties of the omniscient market and simply seeks to fashion ways to make individuals, communities, systems and organizations fit for rigors of the catastrophe yet to come’ (Dean, 2014: 161). The notion of resilience thus entails an ultimate acceptance of the view that the world can neither be changed, nor mastered; hence, the only rational strategy for survival is to adapt to externally imposed changes (Joseph, 2013; Walker and Cooper, 2011).

This neoliberal regime of governance operates a powerful ideological shift of responsibility. Failure, poverty, marginalization and exclusion are no longer the responsibility of governments as global economic and financial processes are beyond the capacity of states to manage them. Individuals are ultimately responsible for their own successes and failures, which become a result of their own resilience, namely, their capacity to adapt to the neoliberal market-based order. Neoliberalism as a governmental rationality based on the construction of resilient subjects insulates neoliberalism from potential attacks and contributes to its very resilience. The crises of neoliberalism are no longer the outcome of a governmental regime, a political philosophy and economic system based on logics of inequality, accumulation and dispossession that should be opposed, but rather the outcome of externally given conditions emerging from the complexity of the neoliberal order.

This approach has the merit of integrating biopolitics into the analysis of the resilience of neoliberalism. In line with Foucault’s (2010: 22n) idea of neoliberalism ‘as the general framework of biopolitics’, this view rests on an understanding of biopolitics as primarily about the production of subjectivity (Hardt and Negri, 2009). Accordingly, the primary goal of biopolitical power is ‘to incite, reinforce, control, monitor, optimize, and organize the forces under it’ in order to integrate the population into neoliberal ‘systems of efficient and economic controls’ by prompting the ‘the adjustment of the accumulation of men to that of capital, [and] the joining of the growth of human groups to the expansion of productive forces’ (Foucault, 1978: 136, 139, 141). Biopolitics thus concerns the production of entrepreneurial and strategizing subjects that, confronted with a world that cannot be changed, are normalized into the acceptance of resilience as the only possible rational course of life. This results not just in their inscription into mechanisms of neoliberal competition, accumulation and inequality, but in their construction as neoliberal subjects, whose resilience is ultimately the resilience of the neoliberal order.

Although powerful and insightful, this perspective rests on a narrow understanding of biopolitics as a process of construction of subjectivities instrumental for the reproduction of neoliberalism. As I shall discuss in the next section, biopolitics also encompasses logics of care and racism that govern the divide between who must live and who must die and that can be considered independent sources of governmental rationality. In order to advance this argument, in the next section, I will first explore the relationship between neoliberalism and biopolitics and then how biopolitical rationalities may intervene in neoliberal dynamics and contribute to governing the resilience of neoliberalism.

## The biopolitical governance of neoliberalism

Foucault’s account of the relationship between neoliberalism and biopolitics is rather puzzling. In his 1978–1979 lectures at the Collège de France, *The Birth of Biopolitics* (Foucault, 2010), Foucault argues that neoliberalism represents a new governmental rationality based on the principle of minimum government and the self-limitation of governmental reason. It marks the emergence of a new form of subjectivity revolving around the notion of *homo oeconomicus* and the reconstitution of society around the concepts of market, competition and enterprise. Liberalism and neoliberalism, Foucault (2010: 22n) contends, should be understood ‘as the general framework of biopolitics’. However, despite this statement and the very title of his lectures, Foucault hardly mentions, let alone explores, the concept of biopolitics. The latter had been introduced in his 1975–1976 lectures, *Society Must be Defended* (Foucault, 2003), and in *The History of Sexuality: Volume 1* (Foucault, 1978 [1976]). Here, Foucault characterizes biopolitics as a transformation in the logic of power, from the traditional sovereign power to kill and let live, to the biopolitical power to make live and let die. The main goal of biopolitics, Foucault contends, is to ‘take care’, namely, to ensure the growth, expansion and flourishing of life under power’s control. Yet, in this rendering of biopolitics, Foucault does not mention the notion of neoliberalism at all. This leaves the question open as to how the relationship between neoliberalism and biopolitics should be understood.

In order to address this ‘unsolved puzzle’ (Lemm and Vatter, 2014: 5), I propose to start with Foucault’s account of neoliberalism and how it differs from classical liberalism.^1^ Classical liberalism is linked to the 18th-century emergence of political economy. This should be understood as a ‘method of government that can procure the nation’s prosperity’ and as a ‘general reflection on the organization, distribution, and limitation of powers in a society’ that enthrones the market as the ‘site of the formation of truth’, that is, as a measure of the efficacy and validity of governmental action (Foucault, 2010: 13, 30). Liberal political economy maintains that the market obeys ‘natural’ and ‘spontaneous’ mechanisms that the government cannot understand in their totality. These mechanisms contribute to the simultaneous maximization of individual and collective value. Adam Smith’s *invisible hand* — the idea that the interests of society are best served by the pursuit of self-interest by rational individual agents in a market free from state intervention — is the paradigmatic illustration of this liberal perspective. For Foucault, the metaphor of the invisible hand signals a profound transformation in the meaning and scope of sovereign power. It suggests that for the sovereign, it is impossible to have ‘any form of overarching gaze which would enable the economic process to be totalized’ (Foucault, 2010: 280).

It follows that the sovereign is ontologically ‘ignorant’ about the economy and it is precisely this ignorance — and the resulting idea that he should not attempt to ‘pursue the collective good’ — that makes possible individual and collective growth (Foucault, 2010: 280–281). Accordingly, with liberalism, the market emerges as a self-organizing entity capable of achieving an equilibrium that maximizes both individual and collective interests and requires a limitation of governmental activity. The market thus becomes a model of government or, as Foucault (2010: 33) puts it, ‘a site of veridiction for governmental practice’. This means that the measure of liberal governmental activity is no longer legitimacy or illegitimacy, but economic success or failure. Finally, in the liberal governmental order, the ‘subject of rights’, or what Wendy Brown (2015) calls *homo politicus*, is complemented by a new emerging form of subjectivity, *homo oeconomicus*, the rational and self-oriented individual who seeks the pursuit of his interests in the market through ‘truck, barter and exchange’ (Smith, 1957 [1776]: 12). Liberalism thus rests on a clear demarcation between the sphere of politics, where the individual is ruled as a ‘subject of rights’, and the economic sphere, where he is governed as a ‘subject of interests’ (Foucault, 2010: 273–276).

Neoliberalism inherits many ideas from liberalism — including the notion of self-regulating markets and the related principle of the self-limitation of governmental activity — but also introduces three key innovations. First, the market is not a natural given revolving around dynamics of exchange, but an artificial and fragile environment centred on logics of competition. Accordingly, the market requires ‘an active governmentality’ (Foucault, 2010: 121), that is, an interventionist state, to support and protect its existence and proper functioning. For Foucault (2010: 121), in the neoliberal order, ‘one must govern for the market, rather than because of the market’, to the effect that the market becomes the all-encompassing framework of meanings and significations that reshapes the sense and purpose of state and society. It follows that the task of governments is ‘to intervene on society as such, in its fabric and depth … so that competitive mechanisms can play a regulatory role at every moment and every point in society’ in order to achieve ‘a general regulation of society by the market’ (Foucault, 2010: 145). The thrust of neoliberalism as a governmental rationality is letting individuals govern themselves by governing the overarching framework of meanings and practices in which individuals operate — hence, by governing the ‘conduct of conduct’ (Foucault, 1991) through the normalization of the principle of market competition.

Second, and accordingly, neoliberalism engenders a process of ‘economization of society and politics’, whereby the ‘exercise of political power’ is ‘modelled on the principles of a market economy’ (Foucault, 2010: 131), and the market, understood as ‘an order of normative reason’ (Brown, 2015: 16, 14), acquires a ‘power of formalization for both the state and society’ (Foucault, 2010: 117). A crucial implication of this process is that speculation and risk are no longer confined to the market, but become general orientations of life. This is reflected in a profound transformation of subjectivity. *Homo oeconomicus* is no longer confined to the sphere of the market, as in classical liberalism, but occupies all spheres of human existence. Neoliberal life thus turns into a speculative enterprise aimed at maximizing one’s own human capital through logics of competition, accumulation and strategic positioning (Brown, 2015: 17).

Third, this speculative approach to life entails a significant exposure to risk and the possibility of failure (poverty, social exclusion and marginalization). For Foucault, the risks stemming from the neoliberalization of life cannot be managed through their socialization. This would run counter to the principles of competition and minimum government, namely, the idea that governments should ‘not obstruct the interplay of individual interests’ because they do not possess any ‘overarching gaze’ that would enable a better pursuit of ‘growth’ (Foucault, 2010: 280). Accordingly, Foucault (2010: 144) contends, ensuring growth is the only way to enable individuals to confront the risks stemming from the neoliberalization of life, as growth, while not ‘providing individuals with a social cover for risks’, accords everyone an ‘economic space within which they can take on and confront risks’. Neoliberalism thus encompasses a greater exposure to risk to the effect that ‘live dangerously’ becomes a defining ethos of neoliberalism (Foucault, 2010: 66, 329).

The critique of resilience discussed in the previous section focuses on this aspect of neoliberalism: ‘individuals are constantly exposed to danger, or rather, they are conditioned to experience their situation, their life, their present, and their future as containing danger’ (Foucault, 2010: 66). Accordingly, individuals are forced to subjectify themselves as entrepreneurial and resilient subjects in order to withstand a neoliberal life loaded with uncertainty and risk and deprived of social protections. Moreover, arguably, the resilience required to confront risk is even greater than that anticipated by Foucault because neoliberalism (partly due to its growing financialization) has proved increasingly unable to generate growth (Brown, 2015: 10; see also Harvey, 2005, 2011). According to the critical scholarship on resilience previously discussed, biopower primarily concerns the construction of entrepreneurial and resilient subjects who may be inscribed in and become part of the neoliberal order.

This important perspective overlooks a central task of biopower: taking care of the population by fostering its flourishing and well-being through a series of technologies that may govern ‘aleatory events’ and ‘dangers’ (Foucault, 2003: 246, 252). Although biopolitics has often been understood as a security apparatus (see, for instance, Dillon and Lobo-Guerrero, 2008), Foucault and subsequent scholarship have not explicitly framed it as instrumental to secure the population from the risks of neoliberalism. Nonetheless, Foucault (2003: 246, 254) argues that biopolitics aims to ‘establish an equilibrium, maintain an average, establish a sort of homeostasis, and compensate for variations within this general population and its aleatory field’ in order to ‘improve life … prolong its duration … improve its chances … avoid accidents, and … compensate for failings’ by installing ‘security mechanisms … around the random element inherent in a population of living beings’. This argument invites us to consider the possibility of biopolitics as a governmental rationality distinct from neoliberalism. Its primary task would be to compensate for the risks and failures engendered by neoliberalism through the establishment of a series of institutions and practices — such as hygiene rules, patterns of saving, consumption and reproduction, health-insurance systems, welfare provisions, pensions, labour standards, women’s and children’s rights, trade unions, education, environmental regulations, financial regulations, and (as I shall discuss in the next section) bailouts of financial institutions at times of crisis — which play a key role in governing the resilience of neoliberalism.

In order to explore this perspective, it is useful to consider the relationship between neoliberalism and biopolitics through Karl Polanyi’s (2001 [1944]) thesis of the ‘double movement’. Polanyi (2001 [1944]) considered that the notion of the market as a self-regulating entity central to the economy was an artificial creation of governments, whose constant intervention was essential to sustain its viability. The notion of self-regulating markets has caused their social disembeddedness, namely, markets act as autonomous entities that increasingly shape social relations. According to Polanyi (2001 [1944]), this has prompted a series of countermovements, primarily engineered by governments, but also by other institutions within society, such as trade unions, focused on the care of the population and its well-being, and thus aimed at the social re-embedding of markets. These countermovements include the introduction of social protections (such as welfare provisions, labour standards, education, health programmes) essential to govern the market economy that, ‘if left to evolve according to its own laws, would create great and permanent evils’ (Polanyi, 2001 [1944]: 136). These evils represent not just moral concerns, but also a problem of survival of the market economy. Leaving ‘the faith of soil and people to the market’, Polanyi (2001 [1944]: 137) observed, ‘would be tantamount to annihilating them’, as this would result, for instance, in the brutal exploitation of labour forces (Guizzo and Vigo de Lima, 2015: 5). Caring for the population and their well-being is essential for the preservation of the market economy, which would otherwise collapse under the weight of its own success and contradictions. The dialectical process of social disembedding and re-embedding of the market is thus, for Polanyi, a defining feature of modern capitalist economy.

There are strong similarities between Polanyi’s and Foucault’s understandings of the market, and between Polanyi’s idea of countermovements aimed at the social re-embedding of the market and Foucault’s notion of biopolitics as a set of technologies of power aimed at governing populations by installing security mechanisms fostering life. Indeed, in a rare and recent attempt to explore the connections between Foucault and Polanyi, Danielle Guizzo and Iara Vigo de Lima (2015: 3) argue that ‘the social disembeddedness of markets claimed by Polanyi had the birth of biopolitical practices as one of its effects’. There is, however, also an important difference between Foucault and Polanyi. Polanyi described the laissez-faire movement aimed at expanding the market and the protective countermovement aimed at ensuring its social re-embedding as opposing forces. In particular, Polanyi (2001 [1944]: 138, 136) considered that countermovements expressed ‘the principle of social protection aiming at the conservation of man and nature’ and, as such, they were ‘incompatible with the self-regulation of the market, and thus with the market system itself’. For Polanyi, the double movement epitomized an underlying tension between market and life. For Foucault, neoliberalism and biopolitics mark an overcoming of this tension: the former through a process of economization of the political and the social domain; the latter through the production of life already imbued with market values (such as competition, productivity and entrepreneurship).

Accordingly, a joint Foucauldian–Polanyian reading suggests that the goal of biopolitical countermovements may be the protection not just of life, but of *neoliberal life*, namely, a life that is already inscribed in and draws its meaning from the neoliberal market. Protecting life would thus require protecting the neoliberal market, which is the condition of possibility of life. This life–market compenetration suggests that the neoliberal disembedding of the market and biopolitical countermovements should be considered complementary rather than competing rationalities of government, and that biopolitical countermovements/governmental rationalities may play a key role in governing the resilience of neoliberalism. This interaction brings to the fore a perspective that differs considerably from those discussed in the previous section. It highlights a different goal of governmentality that transcends purely neoliberal rationalities: not just ‘saving the market’ (Harvey), or ‘governing the system’ (Konings), but governing neoliberal life, namely, ensuring the resilience of neoliberalism by securing neoliberal life through a series of countermovements/biopolitical interventions that transcend the mere construction of resilient subjects.

To better appreciate the role of biopolitics in governing the resilience of neoliberalism, it is worth considering a potential counterargument, namely, neoliberalism’s touted dismantlement of the quintessential biopolitical security apparatus: the welfare state. However, those analyses that lament a shift from public pensions, universal insurance and pay rises to private investments in pension funds, individual insurance and consumer credit (Lazzarato, 2015: 60) overlook how these trends have been met by a series of Polanyian countermovements aimed at reintroducing biopolitical mechanisms of care of the population. The thesis of the ‘compensatory state’ (Glenn, 2007, 2009), for instance, maintains that neoliberalism has resulted not in the shrinking of governments, but in their expansion (measured in terms of social expenditures) in order to ‘compensate’ for the negative effects of neoliberal globalization, such as growing competition and the mobility of capital, the race to the bottom in labour standards, labour flexibility, and unemployment caused by industrial relocations (Glenn, 2007: 132–136). Accordingly, neoliberal ‘[g]lobalization may well require big, not small, government’ (Rodrik, 1996: 26) in order to provide both active labour market policies — such as ‘retraining programs, public employment services, targeting of problematic populations such as school dropouts’ — and passive social programmes — such as employment protections, unemployment benefits, and retirement policies (Spilerman, 2009: 78). The ‘compensatory state’ thesis highlights how the growing uncertainty and risks caused by the process of neoliberal globalization has pushed societies to demand and receive ‘a larger government role as the price of exposing themselves to greater amounts of external risk’ (Rodrik, 1997: 65). This strengthens ‘political incentives for governments to use the policy instruments of the state to mitigate market dislocations by redistributing wealth and risk’ (Garret, 1998: 789; see also Glenn, 2007: 134). This compensatory dimension should be understood not as a complete biopolitical offsetting of neoliberal policies, but as a way of smoothing and governing the harshest effects of neoliberalism, and thus as a way of governing the resilience of neoliberalism by preserving neoliberal life.

Of course, it must be stressed that the compensatory state thesis primarily applies to wealthy industrialized countries. Developing countries have, in fact, witnessed a significant decline in their welfare expenditures (Glenn, 2009), which is indicative of their lower capacity to govern neoliberalism. This North–South divide may suggest that the resilience of neoliberalism in the South is primarily the outcome of an external imposition. This raises the more general question as to whether the biopolitical governance of neoliberalism enacted by the wealthiest and more powerful states — whether through welfare expenditures or other measures, such as the bailout of financial institutions, as I shall discuss in the next section — may result in a ‘transfer’ or ‘projection’ of neoliberal policies to less wealthy countries. This possibility needs to be considered in relation to the very ambivalence of biopolitics. As Foucault (2003: 254) observes, biopolitics is not an unqualified attempt to care for the wealth and well-being of people, but the specific endeavour to promote the life and well-being of the population under power’s control. This care also requires protecting the population from other populations — the ‘inferior species’, to use Foucault’s (2003: 255) terminology — that threaten its possibility to flourish, proliferate and expand. According to Foucault (2003: 254), then, biopolitics inscribes racism ‘in the mechanisms of the State’, turning it into an essential mechanism of state power.

The modern biopolitical racism described by Foucault differs from traditional notions of racism that ascribed negative qualities to certain ethnic groups and portrayed them as inferior. Indeed, modern biopolitical racism ‘is not merely an irrational prejudice, a form of socio-political discrimination, or an ideological motive in a political doctrine; rather, it is a form of government that is designed to manage a population’ (Rasmussen, 2011: 34). It is a biopolitical governmental rationality that introduces ‘a break into the domain of life that is under power’s control: the break between what must live and what must die’ (Foucault, 2003: 254). In this biological framework, Foucault (2003: 255) reminds us:

[t]he fact that the other dies does not mean simply that I live in the sense that his death guarantees my safety; the death of the other, the death of the bad race, of the inferior race … is something that will make life in general healthier: healthier and purer.

In the next section, I will expand this analysis of biopolitical racism with reference to the governmentality of the Greek sovereign debt crisis. In particular, I will discuss how biopolitical rationalities of care and racism have been deployed to govern the Greek crisis and the resilience of neoliberalism, namely, by preventing the crisis from undermining the stability of the European banking system and, therefore, the neoliberal life, wealth and well-being of the European population.

## The biopolitical governance of the Greek sovereign debt crisis

As has been observed (Tsoukala, 2013), two main narratives have characterized the popular debate on the causes of the Greek sovereign debt crisis. The first has focused on the profligacy of the Greek population, the Greek state’s incapacity to comply with the requirements of the European Stability and Growth Pact, and its irresponsibility for falsifying its economic, financial and fiscal situation in order to join the European Union (EU). The second narrative has concentrated on the structural limits of the EU, which has contributed to create trade imbalances and, at the same time, prevent its nations from pursuing an independent monetary policy, such as devaluation, that in the case of Greece, would have helped to govern some of the effects of the crisis (Tsoukala, 2013). The biopolitical racist narrative of the Greeks as a lazy, incompetent and greedy population (see Arnade, 2015), which, according to Angela Merkel, should have not been allowed into the EU in the first place (*The Guardian*, 2010), has emerged as the dominant one. This narrative has portrayed the Greeks as responsible for their own condition, for threatening the stability of the EU and the well-being of its population, and for posing ‘as big a risk to the global economy and financial markets as the collapse of Lehman Brothers did in September 2008’ (*The Guardian*, 2010). For the Organization for Economic Cooperation and Development (OECD) Secretary General Ángel Gurría, the Greek debt crisis was spreading ‘like Ebola. When you realise you have it you have to cut your leg off in order to survive’ (*The Guardian*, 2010). Fear that the ‘contagion’ of ‘the developed world’s most crooked economy’ lying ‘in the cradle of Western civilization’ could trigger a ‘domino effect’ that could undermine ‘the very existence of the world’s second biggest currency’ (see Tracy, 2012) amplified calls for resolute political interventions.

This powerful narrative of biopolitical stigmatization has been instrumental in justifying the adoption of a series of bailouts conditional upon the implementation of austerity measures as a solution to the crisis. In particular, the construction of the Greeks as guilty and therefore deserving of punishment (Engdahl, 2015) has contributed to overshadowing the fact that the primary goal of the three bailouts received by Greece was saving large European banks that owned most of the Greek sovereign debt, rather than helping the Greek economy recover. Since 2010, European governments, the European Central Bank and the International Monetary Fund (the so-called Troika) have loaned Greece €252 billion in order to rescue it from default. However, it is estimated that only 5% of the bailout loan has gone to the Greek government to invest in the country and benefit the Greek people (Rocholl and Stahmer, 2016). The majority of the loan has been used to pay off debt with primarily German and French (and, to a less extent, British, US, Dutch and Italian) banks, which were exposed for a total of over €200 billion following the extensive purchase of Greek sovereign debt in order to reap the higher yields on bonds compared to that offered by other countries in the Eurozone. At the end of 2014, the exposure of European and US banks to Greece had shrunk to about €35 billion (Nardelli and Merler, 2015; Rocholl and Stahmer, 2016), while Greece had plunged into an even deeper recession, with 25% unemployment, over 44% of the Greek population with an income below the poverty line, a 35% increase in suicides and ‘the collapse of public health and education’ (Hadjimichalis, 2014: 505).

The narrative of Greek irresponsibility has contributed to overshadowing a key cause of the crisis: irresponsible lending. As discussed in the previous section, an essential component of neoliberal competition is the acceptance of the risks and dangers that competition may entail, to the extent that, for Foucault (2010: 66), ‘live dangerously’ may well be considered the motto of neoliberalism. The Greek bailout, however, relieved private actors (the banks) of the business risk that they had deliberately and voluntarily undertaken by shifting the risk and its consequences onto the Greek people — and, to a lesser extent, onto the European people in the case of insolvency of the Greek state. By purchasing Greek bonds, European banks were able to benefit from the higher interest rates in the Eurozone while not paying any price for the risk undertaken (a positive relationship between risk and expected return is, in theory, a central principle of financial markets; see, for instance, Chance and Brooks, 2015; Markowitz, 1952; Merton, 1973). Their lending was reckless and perfectly rational as it exploited the idea that an EU country would not default as the EU would ultimately come to its rescue (see Arnade, 2015).

Following Harvey (2005: 29), it could be argued that the fact that lenders, despite their reckless and irresponsible behaviour, are spared losses whereas ‘borrowers are forced by state and international powers to take on board the cost of debt repayment no matter what the consequences for the livelihood and well-being of the local population’ highlights how neoliberalism has been able to ‘capture’ sovereign power and use it to solve its contradictions and govern its resilience. From this perspective, the Greek crisis is only the last of hundreds of crises around the world that have been dealt with by privatizing profits and socializing risks, thus contradicting the neoliberal assumption of self-regulating markets and resulting in a systemic moral hazard (banks are not responsible for their risky behaviours). Following Konings, the Greek bailout can also be considered as part of a pattern in which governments step in to bail out financial actors. However, Konings would regard this intervention as an expression of the very neoliberal duty of states to support the market when mechanisms of self-regulation fail in an economic environment in which governments cannot transcend risk (and therefore prevent crises) solely through regulations. The Greek debt crisis is a case in point: despite the economic, financial and fiscal requirements that Greece had to comply with to join the EU, these were circumvented (with the help of investment bank Goldman Sachs), thus laying the foundations for the recent crisis. According to Konings (2016: 274), when confronted with a crisis whose risk is incalculable, neoliberal governmentality turns the state into a speculative agent that embraces a decisionist — ‘Schmittian’ — understanding of sovereign power in order to preserve the financial system. It suspends the rule of law and, indeed, the very law of the market, and imposes the socialization of private risk/losses, thus guaranteeing the resilience of neoliberalism.

Although insightful, these accounts raise an important objection: is it possible to understand the Greek bailout — and the resilience of neoliberalism — solely as the outcome of a neoliberal governmental rationality that mobilizes sovereign power (either ‘captured’ by neoliberal elites or ‘economized’ by neoliberal rationalities) to save financial institutions? In particular, Konings offers a more nuanced and sophisticated approach than Harvey by arguing that governments have not been ‘captured’ by economic and financial elites, but subjectified as economic agents, which leads them to adopt a speculative attitude (purchasing toxic assets, i.e. Greek debt from the banks) in order to preserve the neoliberal system. However, while this argument explains the governmental rationality of the bailout, it also neglects its underlying rationality of power, which, for Harvey, is crucially represented by the ruling elites using the state to transfer the cost of their reckless behaviour onto the population. The approach that I have been advancing in this article strives to move beyond both Konings’s and Harvey’s by embracing the Foucauldian idea of the economization of the state, yet from the perspective of a biopolitical governmental logic (reconsidered in Polanyian terms) concerned with the care and well-being of the population. From this angle, the rationale of the bailout was not just ‘saving the banks’ on behalf of the ruling economic and financial elites or ‘governing the system’, but governing neoliberal life by saving European citizens from the risk of financial instability caused by the European banks’ exposure to Greek sovereign debt. At the heart of the bailout, in other words, there was biopolitical care for the life of the European population and biopolitical racism towards the Greeks, implicitly constructed as non-Europeans and deemed as a threat to the life and well-being of the Europeans.

The dominant narrative was that the Greeks had lied about their economic and financial credentials, overindulged in welfare spending (including ‘lavish pensions’) and eventually infected with their toxic debt the ‘monetary and financial lifeblood of the [European] economic body’ (see Langley, 2015: 68). The metaphor of money, finance and, more generally, capital as ‘the lifeblood that flows through the body politic of all those societies we call capitalist’ (Harvey, 2010: vii) draws on a deep-seated social imaginary that dates back to Aristotle (Langley, 2015: 68) and helps to understand how the financial threat was ultimately a biological threat. By infecting the bloodstream of Europe, Greece also infected its heart, namely, the banks, endowed with the task of pumping blood/money. The Greek crisis thus threatened to slow down or possibly even suspend the flow of blood, prompting a full-blown crisis of neoliberal capitalism *and* neoliberal life.

This argument highlights the biopolitical centrality of banking to the life, security and well-being of the population. As Paul Langley (2015: 81) observes in relation to the 2008 financial crisis, this was conceptualized and governed not as a crisis concerning the functioning, reliability and efficiency of the market, but primarily ‘as a problem of solvency of banking’. Accordingly, the main goal was not to reform the market, but to restore circulation in order to allow the continuation and reproduction of the neoliberal ‘financialized life’ (Langley, 2015: 98). The ‘affective’ compenetration of banking and life, Langley continues, was highlighted by a number of leading politicians and bureaucrats. Hank Paulson, then US Secretary of the Treasury, warned about the link between ‘a frozen financial system’ and the American people’s ‘reduced values in their retirement and investment accounts’ (cited in Langley, 2015: 98). Gordon Brown, then UK Prime Minister, was even more explicit:

[b]anks aren’t just economic entities, they are woven into the fabric of all our lives, vital to savers, to mortgage holders, to businesses and to ordinary families everywhere. And this isn’t abstract, this is about the conversations mothers and fathers will be having on their sofas tonight once they have put their children to bed. (Cited in Langley, 2015: 98)

Like the 2008 financial crisis, the Greek sovereign debt crisis was conceptualized and governed ‘as a problem of solvency of banking’. Unlike the 2008 financial crisis, there was no need to publically stress the banking–life connection in order to justify the bailout of private companies with public money. The possibility to identify a clear biopolitical cause and threat, the Greek population, provided legitimacy for a punitive bailout that established a biopolitical connection between the need for Greece to embrace austerity and the survival and well-being of the European population.

This perspective challenges the idea that the Greek bailout, instrumental for governing the resilience of neoliberalism, was purely a matter of neoliberal governmental rationality that had either ‘captured’ or ‘economized’ sovereign power. It suggests that a complementary governmental rationality, biopolitics, prompted European states to rescue the banks in order to secure the life of the European population by transferring inequality, poverty and social devastation to the Greek people. Accordingly, the Greek bailout saw political power, that is, European states, acting as biopolitical agents of mediation, coordination and regulation that intervened to govern a neoliberal crisis. To reprise a previous quote by Foucault (2003: 246), European governments intervened to ‘establish an equilibrium, maintain an average, establish a sort of homeostasis, and compensate for variations within this general population and its aleatory field’. Ultimately, it was not just neoliberalism, but a combined neoliberal–biopolitical apparatus, that introduced ‘a break into the domain of life that is under power’s control’: the break between Europe — which had to continue to flourish, proliferate and expand — and the Greek people — left with the social devastation of an overwhelming debt. While my argument suggests a convergence between neoliberalism and biopolitics — bailing out the banks secured the market *and* the population — this should not obscure that the bailout represents a Polanyian ‘countermovement’ aimed at re-embedding and partially offsetting the potentially destructive effects of the neoliberal market in order to ensure the preservation and continuation of (neoliberal) life. Hence, whereas Konings deems bailouts solely as the result of governmental rationalities internal to neoliberalism, which prompt states to turn into speculative actors, my contention is that they should also, if not primarily, be considered an instantiation of biopolitical rationalities of care and racism.

This analysis highlights the centrality of biopolitical racism for the resilience and reproduction of neoliberal life to the effect that biopolitical racism should be understood as a governmental rationality central to the biopolitical-neoliberal apparatus of governance. As Nobel prize-winning economist Paul Krugman (cited in Trumbo and Buxton, 2016) remarked: ‘The drive for austerity [imposed upon Greece] was about using the crisis, not solving it’. Biopolitical racism was not just a ‘response’ to the crisis, but an opportunity to further consolidate neoliberal forms of governance though practices of neoliberal dispossession. Thus, as part of the bailout deal, Greece has had to agree to a privatization memorandum^2^ that commits it to ‘sell off €50 billion in public assets’ — from airports to ports, from landscapes to service utilities such as railways and water supply networks — in order to raise cash to repay his debts (Rankin and Smith, 2015). This controversial plan reflects not just the European belief of Greek guilt, which requires Greece to renounce its material wealth to amend its sins,^3^ but also a neoliberal belief in the benefits of privatization as an effective way of reducing public debt and ‘increasing the efficiency of companies’ and ‘the competitiveness of the economy’ (European Commission, 2015).

However, as a result of the privatization process, Greece has sold most of Piraeus, Greece’s largest port, to Chinese state-owned company Cosco. Similarly, Greece’s 14 most profitable regional airports have been sold to German airport operator Fraport, majority owned by the German state. Hence, privatization has not meant the transfer of public assets to private owners, but the dispossession of Greek public assets by foreign capital and the transfer of wealth from Greece to other countries. Yet, the most controversial aspect of the memorandum concerns the privatization of water supply networks. Germany has strongly pushed Greece to sell its water utilities precisely at a time when many European and, in particular, German cities, including Berlin, are ‘bringing water services back under public control in frustration at rising prices and declining service delivery’ (Mathiesen, 2015; Trumbo and Buxton, 2016). For Harvey (2005), this double standard reveals a long-standing underlying hypocrisy at the heart of neoliberalism — a gap between theoretical assumptions and their practical implementation — which has been instrumental for turning privatization (specifically, forcing countries experiencing a crisis to let go of their public assets at sale price) into a primary tool of ‘accumulation by dispossession’. From the perspective advanced in this article, Germany’s attitude should be understood not just as hypocrisy, but as an expression of the biopolitical governance of neoliberalism. The care and racism at the heart of this governmental rationality entail preserving the financial system at home and transferring the price of this protection across national boundaries through the creation of an unpayable and inexpiable debt. Portrayed as the outcome of the inner guilt of an ‘inferior species’, debt becomes a biopolitical instrument of governance (see Lazzarato, 2015) that justifies preserving welfare provisions and public services — that is, *life* — at home and disrupting it abroad.

To further appreciate this argument, it is useful to compare Germany’s response to the effects of the 2008 financial crisis on its economy and Germany’s approach to the 2009 Greek crisis. In late 2008, Berlin launched ‘an emergency bailout package of 480 billion euros for German banks’, followed by €115 billion to strengthen companies with financial problems, and two stimulus packages worth €80 billion in total to relaunch the economy through investment in infrastructure, tax breaks and business loans (Bennhold, 2008; *Spiegel*, 2010). This ‘unprecedented large-scale economic investment’, supported by government labour subsidies aimed at avoiding redundancies, was highly successful and resulted in steady growth (*Spiegel*, 2010). Many scholars and public commentators (see, for instance, Mazzucato, 2015) have argued that Athens would require a similar investment strategy, accompanied by a series of structural reforms of the Greek economy. Instead, Germany — whose banks were the most exposed to Greek sovereign debt and were leveraged 32 to 1 (Lehman Brothers was leveraged 31 to 1 just before filing for bankruptcy and ushering in the largest financial crisis since the Great Depression) — forced Greece to adopt a completely different and thoroughly neoliberal approach to the crisis based on austerity measures, which has had the effect of creating more debt and social devastation. This double standard can be considered a manifestation of the biopolitical governance of neoliberalism. This entails mobilizing neoliberalism as a weapon in order to draw a biopolitical line between ‘what must live [flourish, proliferate and expand], and what must die’ (Foucault, 2003: 255).

The analysis carried out in this section suggests three main observations. First, there is an important tension at the heart of neoliberalism, which is also reflected in Foucault’s account. As previously discussed, Foucault claims that neoliberalism is based on the principles of competition and ‘governing for the market’ (i.e. supporting its existence and smooth functioning). However, these principles stand in an irresolvable tension. Accepting the principle of competition would have required not bailing out European banks, which would have resulted in the crash of the financial market. Instead, the principle of competition was sacrificed in order to preserve the stability and viability of the market *and* the neoliberal life of the European population.

Second, and accordingly, the Greek bailout responded to biopolitical rationalities of care and racism as part of a biopolitical-neoliberal apparatus that signals the progressive convergence of life and markets. From this perspective, the bailout can be considered a Polanyian countermovement: a biopolitical-neoliberal manifestation of governance instrumental to preserving the financialized life of the European population and the resilience of neoliberalism through the construction of the Greek population as ‘threatening’, ‘inferior’ and ‘non-European’. This case suggests that the biopolitical governance of neoliberalism may manifest itself not just through the adoption of mechanisms of social protection and welfare spending, but also by preserving the viability of financial institutions, which are essential to the flourishing and well-being of neoliberal life.

Third, the biopolitical governance of neoliberalism is a tool that the wealthiest and more powerful states employ to preserve life at home and disrupt it abroad. Foucault, more than Polanyi, is essential to grasp this point with his notion of biopolitical racism. In fact, whereas Polanyi’s perspective invites us to consider how a process of social re-embedding of the markets is essential to ensuring the resilience of neoliberalism and neoliberal life, Foucault’s approach alerts us to the fact that the biopolitical governance of neoliberalism may result in the projection of the tensions of neoliberalism onto less wealthy and powerful countries and in the creation of new opportunities for neoliberal exploitation. This is the case of Greece, condemned to social devastation by a German-led European biopolitical governance that punishes irresponsible borrowing, but not irresponsible lending, and creates neoliberal opportunities for accumulation by dispossession through a regime of imposed privatizations.

## Conclusion

This article has explored the question of the resilience of neoliberalism. It has argued that neoliberalism and biopolitics should be considered two complementary and mutually reinforcing, but analytically distinct, governmental rationalities, and that biopolitical rationalities of care and racism play a crucial role in governing the uncertainty and risk stemming from the neoliberalization of life and neoliberalism’s inclination to produce crises. The discussion began with a review of existing accounts of the resilience of neoliberalism. In particular, I considered a first group of perspectives that have focused on the distinctive qualities of neoliberalism and how these have enabled neoliberalism either to ‘capture’ or ‘economize’ sovereign power and use it to govern its resilience. I then discussed a second group of scholars who have accounted for the resilience of neoliberalism by focusing on its biopolitical capacity to construct resilient subjects capable of adapting to the uncertainties of neoliberal competition and inequality. In the second section, drawing on and partially challenging Foucault, I showed how biopolitics and neoliberalism represent two complementary governmental rationalities revolving around the principles of, respectively, care and competition. To better appreciate their relationship, I advanced a joint Foucauldian–Polanyian reading of biopolitical countermovements as an expression of a governmental rationality aimed at governing populations through security apparatuses that protect neoliberal life from some of the risks, tensions and failures of neoliberalism, thus contributing to the latter’s resilience.

This theoretical perspective was then employed to analyse the governmentality of the Greek sovereign debt crisis. This approach enabled a novel understanding of the Greek bailout as an act of care aimed at rescuing European citizens from the risks of financial instability stemming from the exposure of European banks to Greek debt. Hence, I explored the resilience of neoliberalism not just as a problem of ‘saving the market’ or ‘governing the system’, but as a question of governing neoliberal life, namely, ensuring the resilience of neoliberalism by securing neoliberal life through a biopolitical intervention (the bailout) that transcended the mere construction of resilient subjects. There is no doubt that the punitive bailout, by forcing the Greek population to embrace resilience, has also been a process of construction of neoliberal subjectivities. However, what matters most from the perspective of this article is that while the bailout trained the Greeks to be more resilient, it also trained Europeans to be *less resilient* as it biopolitically sheltered them from the potential failures of their banks. The bailout thus involved complementary but distinct biopolitical and neoliberal rationalities. Central to this analysis has been an appreciation of biopolitics as a politics of life and care of the population, and of racism towards and punishment of those ‘inferior species’ which threaten its survival and well-being. Hence, the punishment of irresponsible borrowers (the Greek population), but not of irresponsible lenders (the European banks), should be considered not exclusively and primarily as a product of the capacity of neoliberalism to ‘capture’ or ‘economize’ sovereign power, but as an outcome of the biopolitical governance of neoliberalism enacted by the most powerful states (European countries and Germany in particular) through biopolitical racism. The goal was to protect the financialized life of the European population by transferring the costs of neoliberalism’s crisis onto the ‘inferior’ Greek population, thus ensuring the resilience of neoliberalism and creating new opportunities for neoliberal dispossession.

This approach has three main implications. First, it challenges existing accounts of the resilience of neoliberalism by maintaining that biopolitics represents a distinct and complementary form of governmental rationality instrumental to governing neoliberalism — hence, it suggests that the resilience of neoliberalism cannot be reduced to its qualities and that the role of biopolitics in this task cannot be reduced to the production of resilient subjects. Second, it articulates an original reading of Foucault’s largely underexplored relationship between neoliberalism and biopolitics. The approach advanced in this article challenges and partially reverses Foucault’s claim that neoliberalism should be understood ‘as the general framework of biopolitics’ by suggesting that biopolitics should be understood as an independent and complementary governmental rationality that plays a key role in governing neoliberalism and its inclination to produce crises. Third, as shown by the analysis of the Greek bailout, the concept of biopolitical governmentality of neoliberalism complements and advances Polanyi’s notion of countermovements. The biopolitical governance of neoliberalism may result not just in the adoption of mechanisms of social protection (the social re-embedding of the market), but also in the preservation of the viability of financial institutions — essential to secure the flourishing and well-being of neoliberal financialized life — and in transferring the crisis onto less wealthy and powerful countries through biopolitical racism in order to advance the dynamics of neoliberal exploitation. Preaching and imposing neoliberal austerity while benefiting from biopolitical policies aimed at boosting the economy and creating jobs — as in the case of Germany’s attitude towards Greece — should be considered a manifestation of a biopolitical rationality that governs neoliberalism as a weapon in order to make ‘us’ live and let ‘them’ die.

The analysis carried out in this article thus indicates that the main task of sovereign institutions has been not just ‘saving the market’ or ‘governing the system’, but governing neoliberal life. This has entailed caring for the population under its control by restoring the circulation of neoliberal financialized life through the identification of an enemy — an ‘inferior species’, to reprise Foucault’s expression — solely responsible for the crisis. This suggests that in order to understand and possibly challenge the resilience of neoliberalism, it is necessary to consider how it is made possible by rationalities of care that preserve neoliberal life through biopolitical interventions. The latter are virtually inseparable from forms of biopolitical racism that displace the source of risk, uncertainty and dispossession from the neoliberal regime onto ‘inferior’ populations, whose lack of compliance with neoliberal dictates is converted into a threat to our neoliberal survival and well-being that deserves punishment and authorizes further dynamics of neoliberal dispossession. Hence, if it is true, as Ani DiFranco sings, that ‘[e]very tool is a weapon if you hold it right’, it is not enough to critique neoliberalism as a weapon, but also vital to understand how the neoliberal weapon is held and handled, as well as who it is directed at and by whom. The concept of the biopolitical governance of neoliberalism advanced in this article aims to offer a novel perspective from which to explore this question.

## Notes

1. In what follows, my goal is neither to provide an exegesis/critique of Foucault’s arguments, nor to provide the ‘correct’ interpretation of his thought, but to reflect and elaborate on Foucault’s ‘deliberately evasive, elusive and provocative’ views (Joseph, 2013: 41) in order to advance a novel understanding of the relationship between neoliberalism and biopolitics.

2. Available at: http://ec.europa.eu/economy_finance/assistance_eu_ms/greek_loan_facility/pdf/01_mou_annex1_20150730_en.pdf (accessed 1 June 2016).

3. Interestingly, the German word for ‘guilt’ and ‘debt’ is exactly the same: *schuld*.
